# Pathological MAPK activation–mediated lymphatic basement membrane disruption causes lymphangiectasia that is treatable with ravoxertinib

**DOI:** 10.1172/jci.insight.153033

**Published:** 2022-09-08

**Authors:** Harish P. Janardhan, Karen Dresser, Lloyd Hutchinson, Chinmay M. Trivedi

**Affiliations:** 1 Division of Cardiovascular Medicine,; 2 Department of Medicine,; 3 Department of Pathology,; 4 Department of Molecular, Cell, and Cancer Biology, and; 5 Li-Weibo Institute for Rare Diseases Research, University of Massachusetts Medical School, Worcester, Massachusetts, USA.

**Keywords:** Development, Vascular Biology, Cardiovascular disease, Lymph, Mouse models

## Abstract

Lymphangiectasia, an anomalous dilation of lymphatic vessels first described in the 17th century, is frequently associated with chylous effusion, respiratory failure, and high mortality in young patients, yet the underlying molecular pathogenesis and effective treatments remain elusive. Here, we identify an unexpected causal link between MAPK activation and defective development of the lymphatic basement membrane that drives lymphangiectasia. Human pathological tissue samples from patients diagnosed with lymphangiectasia revealed sustained MAPK activation within lymphatic endothelial cells. Endothelial KRAS^G12D^–mediated sustained MAPK activation in newborn mice caused severe pulmonary and intercostal lymphangiectasia, accumulation of chyle in the pleural space, and complete lethality. Pathological activation of MAPK in murine vasculature inhibited the Nfatc1-dependent genetic program required for laminin interactions, collagen crosslinking, and anchoring fibril formation, driving defective development of the lymphatic basement membrane. Treatment with ravoxertinib, a pharmacological inhibitor of MAPK, reverses nuclear-to-cytoplasmic localization of Nfatc1, basement membrane development defects, lymphangiectasia, and chyle accumulation, ultimately improving survival of endothelial KRAS mutant neonatal mice. These results reveal defective lymphatic basement membrane assembly and composition as major causes of thoracic lymphangiectasia and provide a potential treatment.

## Graphical abstract

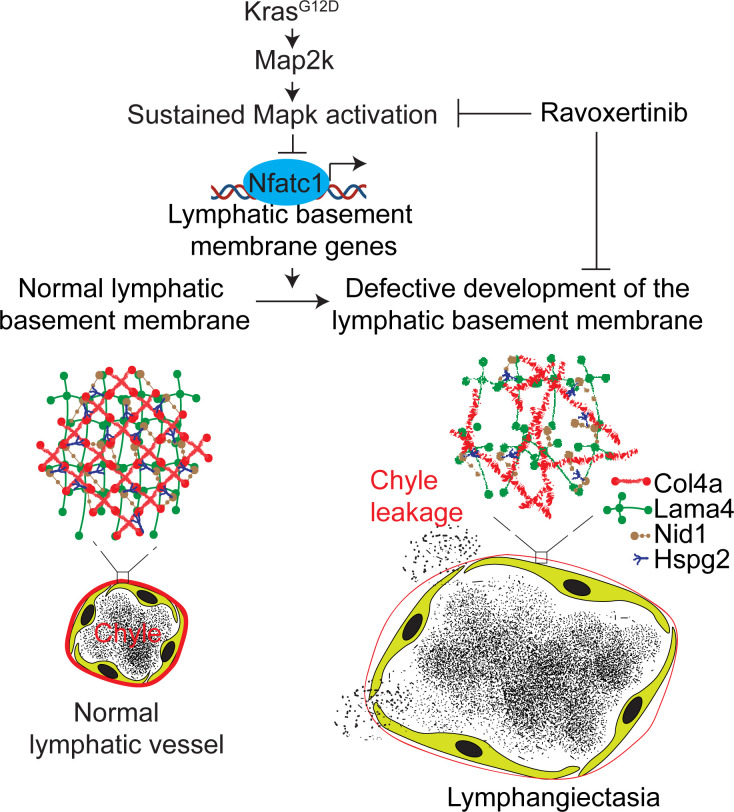

## Introduction

Congenital or neonatal accumulation of chyle in the pleural space is the most common cause of pleural effusion, affecting 1 in 10,000 births with mortality rates between 20% and 60% (1–3). Neonatal patients with a spontaneous accumulation of chyle (lymph with lipoproteins) exhibit bilateral pleural effusion, severe respiratory distress, tachypnea, and cyanosis, suggesting that the mechanical effect of compression on lung compliance and impairment of gas exchange in alveoli (4–8). Accumulation of chyle in the pleural space has long been established as a lymphatic anomaly (9, 10). To facilitate effective gas exchange, an extensive network of lymphatic vessels within the pleura, the intercostal space, the perivascular spaces of arterioles and venules, and the connective tissue of the terminal and respiratory bronchioles collect interstitial fluid (lymph) and drain it back to the venous system (11–14). Abnormal dilation of these lymphatic vessels, known as lymphangiectasia, has been frequently associated with neonatal chylous effusion, immature lungs, and severe respiratory distress with mortality (15–20). Even though Rudolf Virchow described neonatal pulmonary lymphangiectasia as early as 1856 (21), the underlying causal etiology and treatment options remain elusive.

Due to the absence of smooth muscle cells, pulmonary lymphatic vessels rely on the basement membrane to drain lymph, suggesting vulnerability to dysfunction (11). The basement membrane surrounding lymphatic capillaries contains a compact meshwork of interconnected lattice of laminin, collagen IV, and nidogen, which generates a molecular sieve with a pore size of 10 nm (22–24) to facilitate passage of interstitial fluid and macromolecules. Defective basement membrane assembly and/or composition disrupt or enlarge the pore size, causing several human diseases (25–28). Although the basement membrane was first described in 1840 (29, 30), its role in mammalian lymphatic vessel development and disease remains undefined.

In the United States, over 200,000 patients are diagnosed with oncogenic KRAS mutations every year, representing ~75% of RAS mutations — the first human oncogene (31–33). In 1975, Mary Engle et al. described neonatal cases of chylous effusion and primary pulmonary lymphangiectasia associated with Noonan’s syndrome, an autosomal dominant disorder primarily mediated by oncogenic mutations in the RAS/MAPK signaling pathway (34–41). Here, we identify an unexpected causal link between oncogenic MAPK activation and defective development of the lymphatic basement membrane that drives lymphangiectasia in humans and mice. Our studies support a model in which oncogenic mutation KRAS^G12D^ and pathological activation of oncogenic MAPK signaling disrupt lymphatic basement membrane composition and, hence, assembly, causing dilation and increasing pore sizes of pulmonary lymphatic vessels, which in turn promotes chylous effusion in pleural spaces limiting gas exchange in alveoli and neonatal survival. Pharmacological inhibition of MAPK reverses neonatal lymphangiectasia and improves survival of endothelial KRAS^G12D^ mutant mice.

## Results

### Patients with lymphangiectasia exhibit aberrant activation of MAPK signaling in pathological tissue samples.

**Figure 1:**
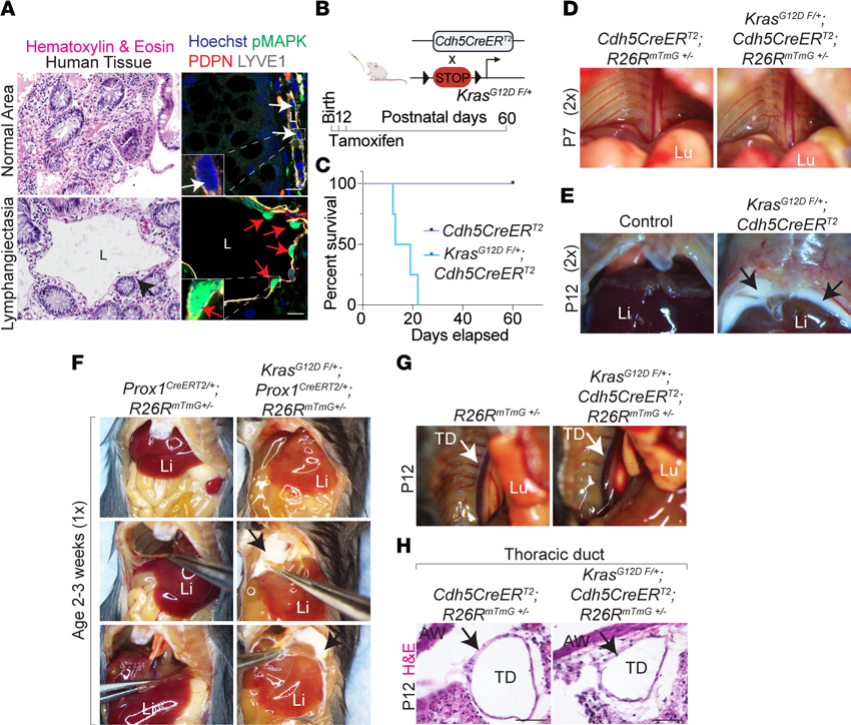
Pathological MAPK activation causes lymphatic dysfunction. (**A**) H&E-stained pathological human tissue section shows lymphangiectasia (black arrows; *n* = 39). Coimmunofluorescent staining with Hoechst nuclear counterstain (blue) shows nuclear localization of phosphorylated MAPK (green) in PDPN^+^ and LYVE1^+^ (red and gray, respectively) lymphatic endothelial cells (red arrows) compared with normal lymphatic vessel (white arrows, *n* = 39). (**B**) Schematic of tamoxifen administration to neonatal mice to activate endothelial expression of Kras^G12D^ allele. (**C**) Kaplan-Meier survival analysis depicting complete postnatal lethality of Kras^G12D^ ^fl/+^; Cdh5^CreERT2^ mice treated with tamoxifen. (**D** and **E**) Dissected thoraces at P7 (**D**) and P12 (**E**) from control and Kras^G12D^ ^fl/+^; Cdh5^CreERT2^ mice. Black arrows show accumulation of chyle in the bilateral pleural cavity in Kras^G12D^ ^fl/+^; Cdh5^CreERT2^ mice treated with tamoxifen at P12. (**F**) Dissected thoraces from control and Kras^G12D^ ^fl/+^; Prox1^CreERT2^; R26R^mTmG+/–^ mice. Black arrows show accumulation of chyle in the bilateral pleural cavity. (**G**) Evans blue dye injection into hind limb footpad of control and Kras^G12D^ ^fl/+^; Cdh5^CreERT2^; R26R^mTmG+/–^ mice at P12 showed normal dye uptake into the thoracic duct (white arrow) and no backflow in the thoracic lymphatics. (**H**) H&E-stained cross section of thoracic duct show normal morphology (black arrow) from control and Kras^G12D^ ^fl/+^; Cdh5^CreERT2^; R26R^mTmG+/–^ mice. Scale bar: 50 μm. All experimental data were verified in at least 3 independent experiments. Li, liver; Lu, lung; TD, thoracic duct.

In patients, emerging data highlight an association between oncogenic mutations in KRAS, including KRAS^G12D^, and lymphatic anomalies (42, 43). Despite this association, the role of KRAS/MAPK signaling in the formation of lymphangiectasia remains unknown. To address this, we analyzed pathological tissue samples from 41 patients diagnosed with lymphangiectasia. Our analyses confirmed lymphangiectasia in 39 pathological samples (Table 1, Figure 1A, and Supplemental Figure 1; supplemental material available online with this article; https://doi.org/10.1172/jci.insight.153033DS1). These samples revealed sustained activation of MAPK signaling within podoplanin (PDPN) and lymphatic vessel endothelial hyaluronan receptor 1 (LYVE1) expressing endothelial cells lining abnormally dilated lymphatic vessels (Table 1, Figure 1A, and Supplemental Figure 1).

### Mice expressing endothelial KRAS^G12D^ oncogenic mutation exhibit chylous pleural effusion and postnatal lethality.

To investigate the endothelial cell–specific role of oncogenic KRAS^G12D^ mutations in lymphangiectasia, we generated Kras^G12D^ ^fl/+^; Cdh5^CreERT2^ mice, in which tamoxifen administration activates the gain-of-function Kras^G12D^ allele specifically in Cdh5^+^ endothelial cells. Kras^G12D^ ^fl/+^; Cdh5^CreERT2^ neonatal mice, treated with tamoxifen at birth, spontaneously exhibited severe respiratory distress, bilateral chylous pleural effusion, and complete lethality between P12 and P20 when compared with controls (Figure 1, B–E). Mice expressing KRAS^G12D^ in Prox1^+^ lymphatic endothelial cells (Kras^G12D fl/+^; Prox1^CreERT2/+^; R26R^mTmG/+^) at birth exhibited a similar phenotype (Figure 1F). Using Evans blue dye, we observed normal anterograde lymphatic flow from the left hind footpad into the thoracic duct and venous circulation in tamoxifen-treated Kras^G12D fl/+^; Cdh5^CreERT2/+^; R26R^mTmG/+^ neonates, suggesting functional integrity of mesenteric lymphatic valves, normal abdominal lymph transport, and an intact thoracic duct (Figure 1, G and H).

### Endothelial KRAS^G12D^ mutation drives pulmonary and intercostal lymphangiectasia in neonatal mice.

**Figure 2:**
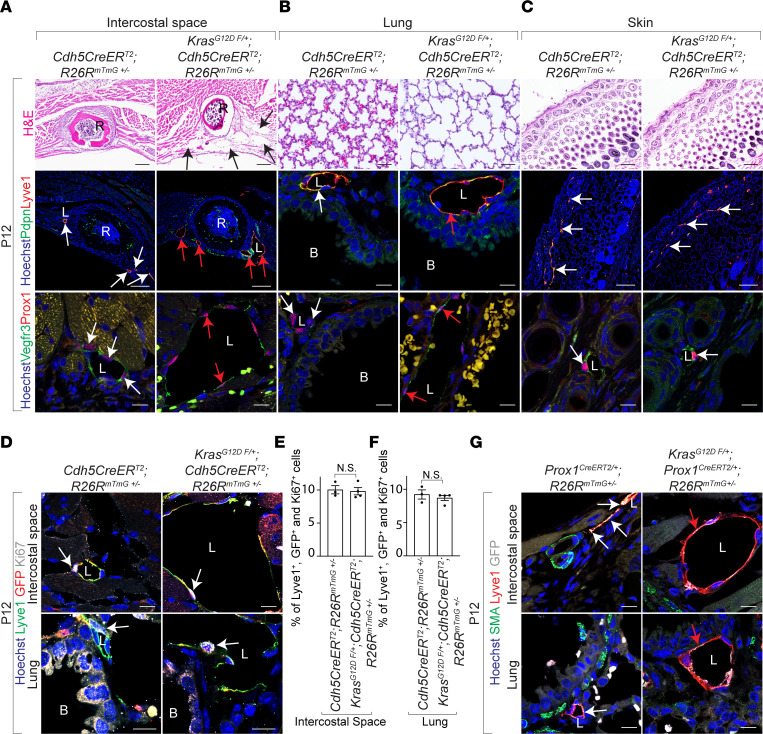
Lymphatic endothelial KRAS^G12D^ mutation drives lymphangiectasia. (**A**–**C**) H&E-stained (upper row), Pdpn (green) and Lyve1 (red) immunofluorescently stained (middle row), and Vegfr3 (green) and Prox1 (red) immunofluorescently stained (lower row) sections of intercostal spaces (**A**), lungs (**B**), and skin (**C**) from control and Kras^G12D^ ^fl/+^; Cdh5^CreERT2^; R26R^mTmG+/–^ mice treated with tamoxifen at P12. White arrows show normal lymphatic vessels. Red arrows show pathological dilation of lymphatic vessels. Black arrows show dilated intercostal vessels. Hoechst nuclear counterstain (blue). Scale bar: 100 μm (top row, middle row [left 2 panels], and middle row [right 2 panels]), 10 μm (middle row [middle 2 panels] and bottom row). (**D**) Lyve1, GFP, and Ki67 immunofluorescently stained sections of intercostal spaces (upper panels) and lungs (lower panels) from control and Kras^G12D^ ^fl/+^; Cdh5^CreERT2^; R26R^mTmG+/–^ mice treated with tamoxifen at P12. White arrows show proliferating lymphatic endothelial cells. Hoechst nuclear counterstain (blue). Scale bar: 10 μm. (**E** and **F**) Quantitation of proliferating intercostal (**E**) and pulmonary (**F**) lymphatic endothelial cells (Lyve1^+^, GFP^+^, and Ki67^+^) in control and Kras^G12D^ ^fl/+^; Cdh5^CreERT2^; R26R^mTmG+/–^ mice treated with tamoxifen (*n* = 3) at P12. Unpaired nonparametric Mann-Whitney *U* test; *P* > 0.3. Data represent the mean ± SEM. (**G**) SMA, Lyve1, and GFP immunofluorescently stained sections of intercostal spaces (upper panels) and lungs (lower panels) from control and Kras^G12D^ ^fl/+^; Prox1^CreERT2^; R26R^mTmG+/–^ mice treated with tamoxifen at P12. White arrows show normal lymphatic vessels. Red arrows show aberrant dilation of lymphatic vessels. Lymphatic vessels lack smooth muscle coverage (green staining). Hoechst nuclear counterstain (blue). Scale bar: 10 μm. All experimental data were verified in at least 3 independent experiments. L, Lymphatic vessel; B, Bronchus; R, Rib.

At P12, histologic and immunologic analyses of tamoxifen-treated Kras^G12D^ ^fl/+^; Cdh5^CreERT2^ neonatal mice revealed aberrantly dilated peribronchial and intercostal lymphatic vessels lined by Pdpn^+^ and Lyve1^+^ or Prox1^+^ and Vegfr3^+^ endothelial cells, enlarged pulmonary alveoli, and normal lymphatic endothelial cell proliferation, consistent with human neonatal pulmonary lymphangiectasia (Figure 2, A–F). Furthermore, we observed peribronchial and intercostal lymphangiectasia in Kras^G12D^ ^fl/+^; Prox1^CreERT2^ neonates treated with tamoxifen at birth (Figure 2G). Consistent with a previous report (11), we did not observe smooth muscle actin expressing cells around Prox1^CreERT2/+^ and Lyve1^+^ intercostal and pulmonary lymphatic vessels (Figure 2G). Lymphatic vessels within skin, heart, and intestinal wall exhibited normal morphology in tamoxifen-treated Kras^G12D^ ^fl/+^; Cdh5^CreERT2^ neonates (Figure 2C, data not shown). These results suggest endothelial gain-of-function KRAS^G12D^ mutation causes pulmonary and intercostal lymphangiectasia, bilateral chylous effusion in the pleural space, defective maturation of the pulmonary alveoli, and complete lethality (Figures 1 and 2).

### Defective assembly of the lymphatic basement membrane in endothelial KRAS^G12D^ mutant mice.

**Figure 3:**
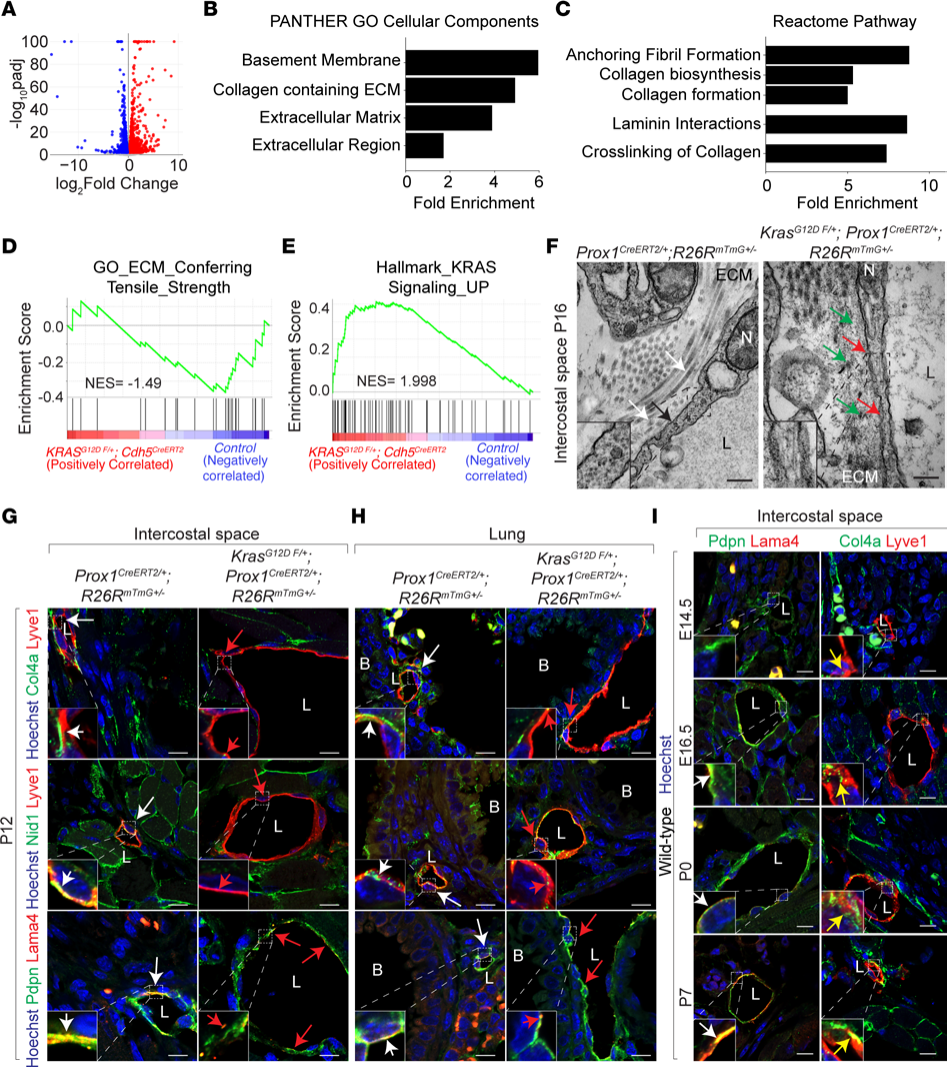
Pathological MAPK activation disrupts lymphatic basement membrane. (**A**) Volcano plot of differentially upregulated transcripts (red), downregulated transcripts (blue), and unchanged transcripts (gray) in P12 Kras^G12D^ ^fl/+^; Cdh5^CreERT2^ lungs treated with tamoxifen compared with control lungs (*n* = 3). (**B** and **C**) Top enriched PANTHER cellular component categories (**B**) and reactome pathway categories (**C**) reveal enrichment in basement membrane categories (*n* = 3). (**D** and **E**) GSEA of differentially regulated transcripts in P12 Kras^G12D^ ^fl/+^; Cdh5^CreERT2^ lungs treated with tamoxifen compared with control lungs (*n* = 3). *P* < 0.05 (**F**) Transmission electron microscopy images of intercostal lymphatic vessels in control Prox1^CreERT2^; R26R^mTmG+/–^ (upper panel) and Kras^G12D^ ^fl/+^; Prox1^CreERT2^; R26R^mTmG+/–^ (lower panel) mice at P16. Red arrows show defective membrane compared with control (black arrow). Green arrows show defective extracellular matrix and collagen compared with control (white arrows). Scale bar: 200 nm. (**G** and **H**) Immunofluorescent staining of intercostal (**G**) and pulmonary (**H**) lymphatic vessels, marked by Lyve1 (red, top 2 rows) or Pdpn (green, bottom row), show reduced expression of basement membrane proteins Col4a (green, top row), Nid1 (green, middle row), and Lama4 (red, bottom row) in sections of Kras^G12D^ ^fl/+^; Prox1^CreERT2^; R26R^mTmG+/–^ mice (red arrows) treated with tamoxifen compared with controls (white arrows) at P12 (*n* = 3). Hoechst nuclear counterstain (blue). Scale bar: 10 μm. (**I**) Immunofluorescent staining for Lama4 (red) and Col4a (green) expression in intercostal lymphatic vessels marked by Pdpn (green) or Lyve1 (red) at embryonic (E14.5 and E16.5) and postnatal (P0 and P7) developmental stages in WT mice. White arrows show Lama4 expression. Yellow arrows show Col4a expression. Hoechst nuclear counterstain (blue). Scale bar: 10 μm. L, Lymphatic vessel; B, Bronchus; ECM, extracellular matrix; N, nucleus. See also Supplemental Figures 2–4.

To determine the underlying transcriptional changes driving the observed phenotype, we performed bulk RNA-Seq analyses of control and tamoxifen-treated Kras^G12D^ ^fl/+^; Cdh5^CreERT2^ lungs derived from P12 mice. Among 2,261 differentially regulated transcripts with National Center for Biotechnology Information identifiers, 49% were upregulated, while 51% were reduced in tamoxifen-treated Kras^G12D^ ^fl/+^; Cdh5^CreERT2^ compared with control (Figure 3A). Reactome pathway, gene ontology, and gene set enrichment analyses (GSEA) showed enrichment in the basement membrane categories containing almost exclusively downregulated genes (Figure 3, B–D, and Supplemental Figure 2), suggesting KRAS^G12D^ inhibits expression of genes required for basement membrane assembly. GSEA also revealed enrichment in KRAS/MAPK signaling target genes, consistent with sustained activation of KRAS^G12D^-dependent signaling pathway and basement membrane categories (Figure 3E). Transmission electron micrographs of endothelial cells lining intercostal lymphatic vessels showed a fragmented basement membrane in tamoxifen-treated Kras^G12D^ ^fl/+^; Prox1^CreERT2^ mice, consistent with failure of basement membrane development (Figure 3F). Consistent with this, we observed corresponding reductions in basement membrane protein expression in pulmonary and intercostal lymphatic vessels, but not in dermal lymphatic vessels of tamoxifen-treated Kras^G12D^ ^fl/+^; Prox1^CreERT2^ mice (Figure 3, G and H; Supplemental Figure 4A; and data not shown). The endothelial basement membrane is composed of 2 polymeric networks of laminin and collagen IV linked by nidogen and proteoglycans (22, 23). The basement membrane exhibits tightly controlled spatiotemporal composition and structure during embryonic development and postnatally (44–46). Several components of the basement membrane are often expressed in a tissue-specific manner to promote development and maturation of the basement membrane (24, 47–49). Supporting this model, analyses of human and murine lung single-cell RNA-Seq data sets revealed robust expression of the basement membrane genes Col4a1, Lama4, and Nid1 in Prox1^+^ lymphatic endothelium (Supplemental Figure 3) (50, 51). Murine pulmonary and intercostal lymphatic vessels exhibited a gradual increase in deposition of Lama4, Col4a, and Nid1 protein from E14.5 to P7, suggesting precise regulation of lymphatic basement membrane composition (Figure 3I, data not shown).

### Pathological MAPK activation represses Nfatc1-dependent transcription of lymphatic basement membrane genes in neonatal mice.

**Figure 4:**
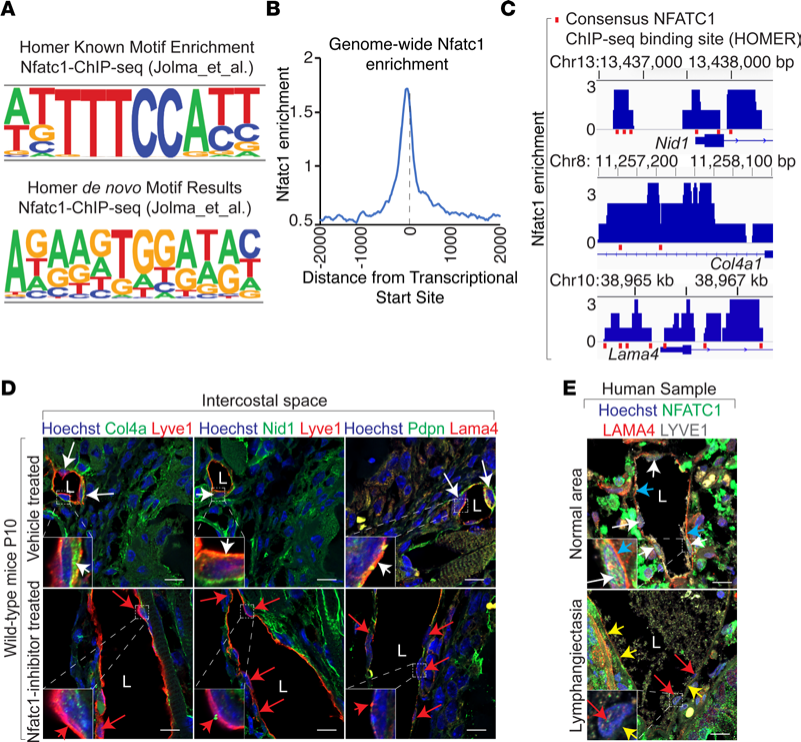
Pathological MAPK activation represses Nfatc1-dependent transcription of lymphatic basement membrane genes. (**A**) Top consensus lymphatic endothelial motif enriched in phosphorylated-MAPK CUT&Tag DNA fragments isolated from KRAS^G12D^ mutant lungs compared with controls. *n* = 3, *P* < 1 × 10^–9^ (known motif), *P* < 1 × 10^–310^ (de novo motif). (**B** and **C**) CUT&Tag analyses of Nfatc1 showing enrichment at transcriptional start sites (**B**) and basement membrane genes Col4a1, Nid1, and Lama4 regulatory loci (**C**) in P12 WT mice. Blue peaks (tracks) represent Nfatc1-occupied DNA sequences. Red boxes represent consensus Nfatc1 binding sites identified by ChIP-Seq (HOMER). (**D**) Immunofluorescent staining for Col4a (green)/Nid1 (green) or Lama4 (red) expression in intercostal lymphatic vessels marked by Pdpn (green) or Lyve1 (red) in P10 WT mice (*n* = 3) treated with vehicle (top row) or Nfatc1 inhibitor (bottom row). Scale bar: 10 μm. (**E**) Immunofluorescent staining of human lymphangiectasia tissue samples (*n* = 7) show decreased nuclear localization of NFATC1 (red arrows) and reduced LAMA4 expression (yellow arrows) compared with nonpathological areas (white arrows). Scale bar: 10 μm.

The Cleavage Under Targets and Tagmentation–based (CUT&Tag-based) genome-wide phosphorylated MAPK occupancy screen revealed enrichment at the consensus Nfatc1 cis-regulatory elements, including lymphatic basement membrane genes in tamoxifen-treated Kras^G12D^ ^fl/+^; Cdh5^CreERT2^ lungs (Figure 4A). Nfatc1, a transcription factor, is required for the maturation and remodeling of the lymphatic vasculature (52). Genome-wide profiling of transcription factor Nfatc1 occupancy revealed enrichment at the regulatory elements of basement membrane genes (Figure 4, B and C). To determine the requirement of Nfatc1 in lymphatic basement membrane development, we administered Nfatc1 inhibitor in WT mice from P3 to P9. These mice exhibited intercostal lymphangiectasia, defective development of the lymphatic basement membrane, and postnatal lethality, consistent with the phenotype of mice expressing KRAS^G12D^ in lymphatic endothelium (Figure 4D, data not shown). Human pathological tissue samples from patients diagnosed with lymphangiectasia exhibit defective lymphatic basement membrane and nuclear-to-cytoplasmic localization of NFATC1 (Figure 4E). Together, these results suggest that NFATC1 functions downstream of the KRAS/MAPK signaling pathway during development of lymphatic basement membrane.

### MAPK inhibition reverses lymphangiectasia and improves survival in endothelial KRAS^G12D^ mutant mice.

**Figure 5:**
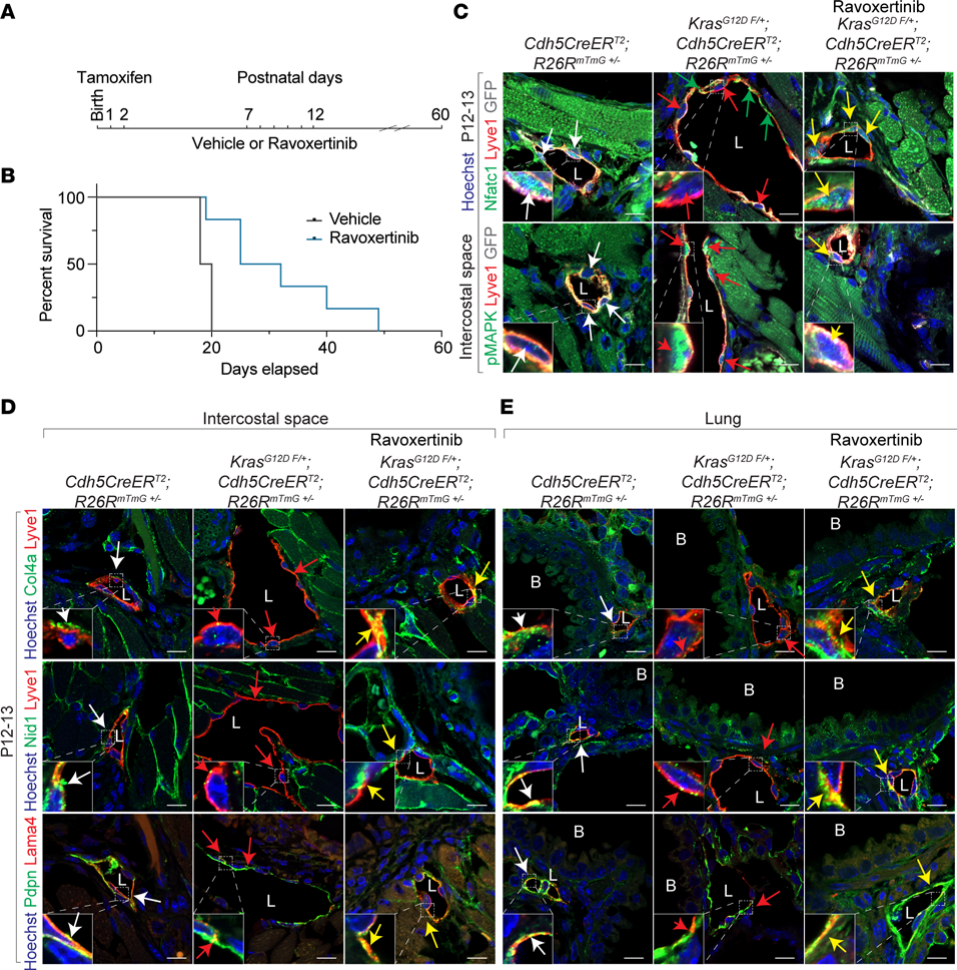
Pharmacological MAPK inhibition reverses lymphangiectasia in endothelial KRAS^G12D^ mutant mice. (**A**) Schematic of vehicle or ravoxertinib administration to neonatal mice to inhibit MAPK activity in endothelial Kras^G12D^ mutant mice. (**B**) Kaplan Meier survival plot depicting improved neonatal survival of Kras^G12D^ ^fl/+^; Cdh5^CreERT2^ mice treated with ravoxertinib compared with vehicle. (**C**) Immunofluorescent staining of intercostal lymphatic vessels, marked by Lyve1 (red), show reduced nuclear expression of Nfatc1 (red arrows, top row) and increased nuclear expression of phosphorylated-MAPK (red arrows, bottom row) in sections of Kras^G12D^ ^fl/+^; Cdh5^CreERT2^; R26R^mTmG+/–^ mice compared with controls (white arrows) and MAPK inhibitor–treated (yellow arrows) mice (*n* = 3). Green arrows show cytoplasmic Nfatc1. Hoechst nuclear counterstain (blue). Scale bar: 10 μm. (**D** and **E**) Immunofluorescent staining for Col4a (green, top row), Nid1 (green, middle row), and Lama4 (red, bottom row) expression in intercostal (**D**) and lung (**E**) lymphatic vessels, marked by Lyve1 (red, top 2 row) or Pdpn (green, bottom row). Yellow arrows show restoration of expression in ravoxertinib-treated mutant Kras^G12D^ ^fl/+^; Cdh5^CreERT2^; R26R^mTmG+/–^ mice similar to control mice (white arrows) and in contrast to untreated mutant mice (red arrows) (*n* = 3). Hoechst nuclear counterstain (blue). Scale bar: 10 μm. L, Lymphatic vessel; B, Bronchus. See also Supplemental Figure 4.

**Figure 6:**
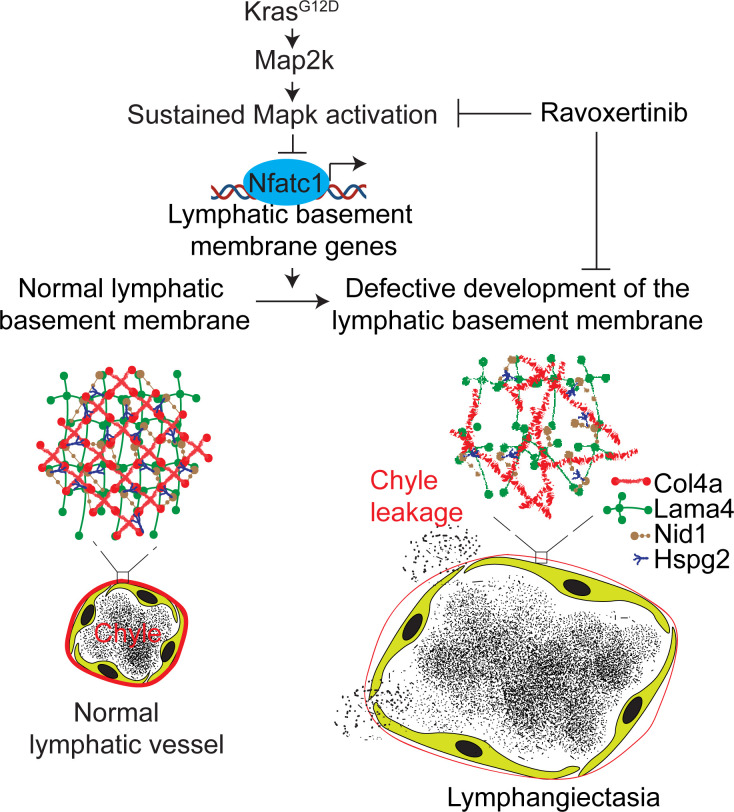
Pathological activation of KRAS/MAPK signaling causes lymphangiectasia. KRAS^G12D^-mediated activation of MAPK pathway represses transcription of basement membrane genes, leading to defective development of lymphatic basement membrane, which in turn promotes pulmonary and intercostal lymphangiectasia, as well as chyle effusion, in the pleural space.

To investigate the requirement of MAPK hyperactivation in the pathogenesis of lymphangiectasia and defective basement membrane development, we administered ravoxertinib, a pharmacological inhibitor of MAPK, from P7 to P12 in Kras^G12D^ ^fl/+^; Cdh5^CreERT2^ mice treated with tamoxifen at birth (Figure 5A). In these mice, inhibition of MAPK activity reversed cytoplasmic localization of Nfatc1 to nucleus, pulmonary and intercostal lymphangiectasia, and defective development of lymphatic vessel basement membrane, and it improved neonatal survival (Figure 5, A–E, and Supplemental Figure 4, A–D). These results identify pathological activation of the KRAS/MAPK pathway as a causal mechanism for defective lymphatic basement membrane leading to pulmonary and intercostal lymphangiectasia and chyle effusion in the pleural space; impairment of gas exchange in the alveoli and subsequent neonatal lethality (Figure 6).

## Discussion

To our knowledge, this report is the first causal link between KRAS mutation and defective development of the lymphatic basement membrane as the driving factors of thoracic lymphangiectasia. The basement membrane, first described in 1840 as membranous sheath in muscle, is present in primitive multicellular organisms, like sponges and *Hydra*, suggesting an essential role of these evolutionarily ancient structures in tissue formation and function (29, 30). Despite this, the roles of the basement membrane in mammalian lymphatic vessel development and disease remain largely undefined. Our findings support a model in which defective assembly and composition of the basement membrane disrupts the tensile strength of the lymphatic vessels, leading to lymphangiectasia (Figure 3). This is consistent with the existing notion that defective basement membrane promotes vessel dilation (47, 53–55). In mammals, a laminin heterotrimeric complex, comprised of α, β, and γ chains, functions as the foundation for the development of the basement membrane (22, 56–58). Specific cell types, including lymphatic endothelial cells, secrete laminin to promote an assembly of a sheet-like lattice, which in turn tightly binds with cell surface receptors, such as integrins (59, 60). Emerging single-cell RNA-Seq studies in both human and murine pulmonary lymphatic endothelial cells have identified cell-specific expression of the basement membrane gene transcripts, including Lama4, Lama5, Lamb1, Lamb2, Lamc1, Lamc2, Col4a1, Col4a2, and Nid1 (Supplemental Figure 3) (50, 51). Collagen IV from these cells also contributes to an independent and covalently crosslinked network, which tethers laminins, nidogen, and integrins together to confer the critical tensile strength of the basement membrane (23, 29, 56, 60, 61). Pathological MAPK activation reduced spatiotemporal expression of basement membrane components, including collagen IV, laminin, nidogen, and proteoglycans, in KRAS^G12D^ mutant endothelial cells lining aberrantly dilated lymphatic vessels, promoting defective development of the basement membrane (Figure 3, G and H). Consistent with this, recent studies revealed the requirement of RASA1, a negative regulator of MAPK signaling, for deposition of collagen IV surrounding lymphatic vessels (62, 63). These data support prior observations that mutations in laminin or loss of collagen IV or nidogen lead to embryonic lethality due to defective development and maturation of the basement membrane (64–69). Cumulatively, these results suggest the physiological MAPK signaling and development of the basement membrane are critical for proper morphogenesis of the thoracic lymphatic vessels and postnatal survival.

Our studies reveal a potentially novel functional role of lymphatic basement membrane in thoracic lymph drainage, an essential process for gas exchange in pulmonary alveoli and neonatal survival. Recent studies demonstrated a requirement of pulmonary lymphatic vessel function to drain pleural fluid not only to prevent edema and inflammation, but also to promote lung inflation and effective gas exchange at birth (11, 70). Due to the absence of smooth muscle cells, pulmonary lymphatic vessels lack the ability to independently drain lymph, suggesting vulnerability to dysfunction (11). Consistent with this observation, we did not observe smooth muscle actin–expressing cells around Prox1^CreERT2/+^ and Lyve1^+^ intercostal and pulmonary lymphatic vessels (Figure 2G). The basement membrane components also recruit growth factors, interact with receptors, regulate cellular proliferation, and provide a permissive environment to support surrounding tissue development and homeostasis (71, 72). Improper basement membrane assembly disrupts development, maturation, and function of vasculature, including lymphatic vessels and surrounding tissues (73). For instance, in Alport’s syndrome, mutations in Lama4, Lama5, or collagen IV genes disrupt or enlarge basement membrane pores leading to severe leakage of plasma proteins into the urine, known as proteinuria (25–28). Similarly, autoantibodies against laminin and collagen IV in Goodpasture’s syndrome disrupt the basement membrane, causing acute kidney failure and lung hemorrhage (25, 74–76). Consistent with this interpretation, KRAS^G12D^ mutation disrupts lymphatic basement membrane assembly and composition, likely causing dilation and increasing pore sizes of thoracic lymphatic vessels, which in turn promotes chylous effusion in pleural spaces, dilating alveolar sacs and limiting gas exchange in alveoli and neonatal survival (Figure 6).

Our findings that pathological activation of MAPK causes lymphangiectasia dovetail with recent studies identifying Noonan’s syndrome patients with chylothorax, lymphedema, and pleural effusion and gain-of-function mutations in components of the MAPK signaling pathway, including *ARAF*, *BRAF*, *NRAS*, *HRAS*, *KRAS*, *PTPN11*, *CBL*, *SOS1*, *RIT1*, *RASA1*, and *SHOC2* (42, 77–88). This is consistent with the high prevalence of lymphatic anomalies observed in a cross-sectional cohort study of patients with Noonan’s syndrome, suggesting an association between the MAPK signaling pathway and lymphatic defects (89). Our study, for the first time to our knowledge, identifies MAPK as an effective pharmacological target for thoracic lymphangiectasia and chylous effusion in mice. Here, we demonstrate that KRAS^G12D^ gain-of-function mutation drives pulmonary and intercostal lymphangiectasia without modulating the rate of lymphatic endothelial cell proliferation (Figure 2, D–F), consistent with Mulliken and Glowacki’s binary characterization of vascular anomalies described in 1982 (90). This study, and previous literature reports from our group and others, further highlight nononcogenic roles of KRAS gain-of-function mutations (91–93). KRAS^G12D^ mutations drive activation of RAF-MAP2K, which in turn phosphorylates and activates MAPK, the effector node of KRAS/MAPK signaling pathway (94). Our studies identify sustained activation of MAPK as a potentially novel transcriptional regulator of lymphatic basement membrane genes, including laminin, collagen IV, nidogen, and Hspg2, required for basement membrane assembly and postnatal maturation. Neonatal mice expressing KRAS^G12D^ in lymphatic endothelium at birth exhibit pulmonary and intercostal lymphangiectasia at P7. Administration of ravoxertinib, an orally bioavailable selective inhibitor of MAPK activity (95), from P7 to P12, not only reversed thoracic lymphangiectasia and defective development of lymphatic vessel basement membrane, but also improved neonatal survival. Consistent with this, ravoxertinib also inhibits growth of KRAS-mutant xenografts in nude mice (95). FDA-approved trametinib and other small molecule inhibitors of MAP2K, an upstream kinase of MAPK, have demonstrated therapeutic promise for the Noonan’s syndrome patients with lymphatic defects (85, 96). However, resistance to MAP2K inhibitors or a negative feedback loop within the KRAS/RAF/MAP2K/MAPK pathway frequently bypass MAP2K and restore sustained MAPK activation (97–99). Hence, targeting the effector node MAPK directly inhibits the KRAS/MAPK signaling pathway, addressing innate or acquired resistance in patients with KRAS mutations (100). Overall, our study identifies MAPK as a potentially novel therapeutic target to treat thoracic lymphangiectasia and chylous effusion, thus improving neonatal survival.

## Methods

### Study design and mice.

Kras^LSL-G12D^ (Kras^G12D fl/+^), Cdh5^CreERT2^, Prox1^CreERT2^, and ROSA26^mTmG^ reporter mice have been described previously (101–104). All mice were maintained on a mixed genetic background. To induce Cre-dependent expression of mutant Kras^G12D^, tamoxifen was orally administered to newborn pups on P0, P1, and P2 at a dose of 50 μg/day and euthanized at P7, P12, or P16 for analyses. For pharmacological treatment, neonatal pups were orally administered with either vehicle solution (30% PEG and 5% Tween 80 dissolved in water) or ravoxertinib (40 μg in vehicle solution) as 2 divided doses about 6 hours apart each day from P7 to P12 and were observed until 2 months of age for survival analyses or alternately euthanized and dissected at P13 for tissue analyses. For Nfatc1 inhibitor treatment, neonatal pups were orally administered with either vehicle solution (10% DMSO and 90% sunflower seed oil) or Cyclosporin A (Selleckchem, 100 μg/day from P3 to P7 and 150 μg/day from P8 to P9 dissolved in 10% DMSO, 90% Sunflower seed oil).

### Patient samples.

After searching the pathology database for diagnostic keyword “lymphangiectasia,” we identified, reviewed, obtained, and verified 39 pathological tissue samples of patients with lymphangiectasia. We have used a data collection sheet to record data using a deidentified, separate numbering system.

### Histology.

Thoraces from neonatal pups were fixed in 2% paraformaldehyde at 4°C for at least 3 days and then decalcified by washing in a fresh solution of 12.5% EDTA, pH 7.0, every 24 hours for 4 days. Subsequently, the tissues were washed in 1× phosphate buffered saline (PBS), dehydrated with a graded series of ethanol and xylene, and embedded in paraffin; finally, the tissue blocks were sectioned at 8 μm thickness using a Leica microtome. H&E staining was performed according to standard methods. Briefly, slides with tissue sections were washed in xylene and ethanol to deparaffinize the tissue section. The sections were then subject to staining with 2 minutes of Harris Modified hematoxylin and 30 seconds of Eosin Y, subsequently dehydrated with ethanol and xylene and mounted with coverslips using Vectashield glass mounting medium.

### Immunofluorescent staining.

Slides with tissue sections were processed for immunofluorescent staining as previously described (105, 106). Briefly, after deparaffinization, the slides were immersed in a chamber containing sodium citrate buffer (10 mM sodium citrate and 0.05% Tween-20, pH 6), and heat-induced antigen retrieval was performed using an antigen retriever (Aptum Biologics) or alternately using a commercial pressure cooker at high setting for 15 minutes. Subsequently, blocking of tissue sections was performed by incubating in a solution of 10% donkey or rabbit serum, 0.1% BSA, and 0.3% Triton X-100 in 1× PBS for 1 hour at room temperature. Samples were then washed 3 times with 1× PBS and incubated overnight at 4°C with primary antibodies (Supplemental Table 1) diluted in 10% donkey or rabbit serum and 1× PBS. Finally, after another 3 washes in 1× PBS, sections were incubated with appropriate fluorophore conjugated secondary antibodies (1:500, Supplemental Table 1) and Hoechst (1:1000) for 1 hour at room temperature. Finally, slides were mounted using coverslips with Vectashield Antifade glass mounting medium.

### Imaging.

Bright-field images of dissected mice and tissue samples were captured using a Leica MZ10 F fluorescence stereomicroscope equipped with a 0.7× C-mount, Achromat 1.0× 90 mm objective, a SOLA light engine, a DS-Fi1 color camera (Nikon), and NIS Elements Basic Research software (Nikon), as described previously (107, 108). H&E-stained sections were imaged using a Nikon Eclipse 80i microscope equipped with CFI Plan Fluor 4×, 10×, 20×, and 40× objective lenses; a DS-Fi1 color camera; and NIS-Elements Basic Research software. Immunofluorescently stained slides were also imaged using Plan-Apochromat objectives with DIC (63×/1.4 oil, and 20×/0.8) on a Zeiss LSM800 Airyscan inverted digital spectral confocal microscope equipped with Definite Focus 2.0; 3 confocal GaAsP detectors, including Airyscan detector with 6000 × 6000 pixels resolution; and solid-state laser module with a 405, 488, 561, and 640 nm beam splitter. Image stacks of vertical projections were assembled using Zeiss Zen 2.5 imaging software.

### Transmission electron microscopy (TEM).

Segments of the mouse rib cage from P16 control and mutant mice were dissected, washed in cold 1× PBS, and immediately fixed in 2.5% glutaraldehyde/1.6% paraformaldehyde in 0.1M sodium cacodylate buffer (pH 7.2). Samples were processed and analyzed at the University of Massachusetts Medical School Electron Microscopy core facility according to standard procedures. Briefly, fixed samples were moved into fresh 2.5% glutaraldehyde/1.6% paraformaldehyde in 0.1M sodium cacodylate buffer and left overnight at 4°C. The samples were then rinsed twice in the same fixation buffer and postfixed with 1% osmium tetroxide for 1 hour at room temperature. Samples were then washed 3 times with ddH_2_O for 10 minutes and then dehydrated through a graded series of ethanol (10%, 30%, 50%, 70%, 85%, 95% for 20 minutes each) to 3 changes of 100% ethanol. Samples were then infiltrated in 50%/50% ethanol/Spurr hard formulation resin mixture. The following day, changes of fresh 100% Spurr resin were performed before the samples were polymerized at 68°C in embedding molds. The samples were then trimmed for TEM. In total, 1 μM semi-thin sections were placed on glass slides and stained with toluidine blue while 70 nm thin sections were placed on gold support grids and contrasted with lead citrate and uranyl acetate. Sections were examined using the Phillips CM10 TEM with 80 Kv accelerating voltage, and images were captured using a Gatan TEM CCD camera.

### Lymphangiography with Evans blue dye.

P12 mice were anesthetized by brief inhalation of isoflurane. The left hind limb footpad was wiped with 70% ethanol and allowed to dry; then, approximately 50 μL of freshly prepared 1% (wt/vol) Evans blue dye dissolved in 1× PBS (pH 7.4) was injected intradermally into the dorsal food pad with the needle (30G) pointed toward the heel. Fifteen minutes after injection, mice were euthanized and the thoracic cavity was opened to observe flow of dye through the thoracic duct and noted for any abnormal reverse flow into the thoracic lymphatic vessels using a Leica dissection stereomicroscope.

### RNA-Seq.

Whole lung from control and mutant neonatal mice at P12 was dissected and briefly washed in cold 1× PBS to clear blood and was immediately snap frozen in liquid nitrogen and stored at –80°C until further analyses. Total RNA isolation from snap-frozen lungs was performed using QIAGEN RNeasy mini kit (catalog 74104) according to manufacturer instructions. Whole lung from each sample homogenized in lysis buffer RLT (Qiagen) provided with the kit and equal aliquots of the lysates from 2 controls and 2 mutants were respectively pooled prior to further downstream steps. RNA was finally eluted with RNase free water, quantified using nanodrop, and sent for sequencing (Novogene) as 3 replicates for each pooled sample. RNA library preparation and sequencing using NovaSeq 6000 PE150 were performed by Novogene Co. Ltd. Raw fastq files were processed using RNA-Seq pipeline of the DolphinNext platform (109). Paired end reads were mapped to mm10 using STAR (110) to generate BAM files, which were then used for transcript quantification using RSEM (111). Using gene-mapped count files from the RSEM output, differential expression analysis was performed in the DEBrowser interface (112).

### CUT&Tag.

Whole lungs were dissected from 3 control and 3 mutant neonatal mice at P12 and briefly washed in cold 1× PBS to clear blood and were immediately snap frozen in liquid nitrogen and stored at –80°C until further analyses. CUT&Tag technique (113) to profile genomic localization of proteins was performed as per manufacture instructions (Active Motif, 53160) with modifications. Briefly, frozen lung tissue was retrieved from –80°C and processed for isolation of nuclei using dounce homogenizer in 1× Swelling buffer (10 mM HEPES, 2 mM MgCl_2_, 3 mM CaCl_2_, 1× Protease inhibitor cocktail in ddH_2_O). The homogenates were incubated for 20 minutes on ice and subsequently passed through a 70 μm cell strainer and centrifuged at 300*g* in 4°C for 10 minutes. The pellet was resuspended in 1× Lysis buffer (10 mM HEPES, 2 mM MgCl_2_, 3 mM CaCl_2_, 10% glycerol, 1% NP40, 1× protease inhibitor cocktail in ddH_2_0), incubated on ice for 5 minutes and centrifuged at 600*g* for 5 minutes at 4°C. The pellet was resuspended in 1.5 mL of wash buffer (Active Motif, catalog 53160) and then passed through a 40 μm cell strainer, and the filtered solution containing nuclei was used for downstream processing. Approximately 100,000 nuclei were resuspended in 1.5 mL wash buffer (Active Motif, 53160) and mixed with 20 μL of Concanavalin A beads (Active Motif, 53160). The bead-bound nuclei were incubated overnight with either 1:10 of IgG antibody (Cell Signaling Technology, 66362), 1:50 of phosphorylated MAPK antibody (Cell Signaling Technology, 4370), or 1:50 of Nfatc1 antibody (Novus Bio, NB100-56732). Further downstream steps were performed according to the protocol provided in the kit (Active Motif, 53160). DNA libraries were prepared using unique i7 and i5 index primers for each sample, size selected with SPRI beads (Active Motif, Catalog No. 53160), quality checked with fragment analyzer, a capillary electrophoresis instrument for next-generation sequencing library quality check, and sequenced by the UMass Deep Sequencing Core with 25 bp paired-end Illumina Miseq system. Paired-end reads were trimmed to 25 bases, barcodes were removed, and reads were aligned to mm10 using Bowtie2 (114) with the parameters -N 1 and -X 1000. Duplicates were removed using Picard tool (115). Low-quality score reads (MAPQ < 10) were removed. These reads were processed in HOMER (116). Genome browser tracks were generated from mapped reads using the “makeUCSCfile” command. Mapped reads were aligned using the “annotatePeaks” command. To identify regions of the genome that exhibited differential occupancy of phosphorylated MAPK in mutant samples compared with controls, we used the ChIPseeker Bioconductor package (117) in the DolphinNext platform (109). As input files, we used called peaks from MACS (114) analysis of control and mutant.

### Data and materials availability.

All data are available in the main text or the supplemental materials.

### Statistics.

Statistical significance was determined based on a nonparametric Mann-Whitney *U* test or χ^2^ test (GraphPad Prism 9.0). *P* < 0.05 was considered significant.

### Study approval.

University of Massachusetts Medical School IACUC approved all animal use protocols. Review of clinical and pathological information and study of archived tissue samples have been approved with a waiver for informed consent (Health Insurance Portability and Accountability Act waiver) by the University of Massachusetts Medical School IRB.

## Author contributions

Conceptualization was contributed by CMT. Methodology was contributed by HPJ, KD, LH, and CMT. Investigation was contributed by HPJ, KD, LH, and CMT. Funding acquisition was contributed by CMT. Supervision was contributed by LH and CMT. Writing of the original draft was contributed by HPJ and CMT. Review and editing were contributed by HPJ, KD, LH, and CMT.

## Supplementary Material

Supplemental data

## Figures and Tables

**Table 1:**
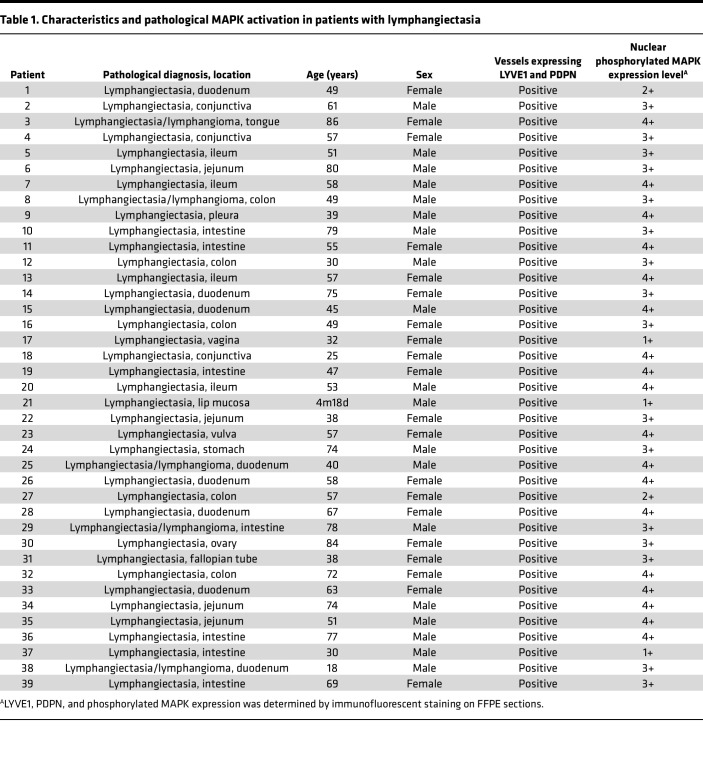
Characteristics and pathological MAPK activation in patients with lymphangiectasia

## Version 1 09/08/2022

Electronic publication
