# Supplementary material for: Pathological MAPK activation–mediated lymphatic basement membrane disruption causes lymphangiectasia that is treatable with ravoxertinib
Source: JCI Insight. 2022 Sep 8;7(17):e153033. doi: 10.1172/jci.insight.153033 (PMC9536262; doi:10.1172/jci.insight.153033)
Supplement: Supplemental data [file jciinsight-7-153033-s056.pdf]

Pathological activation of MAPK causes lymphangiectasia treatable with Ravoxertinib

Authors: Harish P. Janardhan[1,2] Karen Dresser[3], Lloyd Hutchinson[3], and Chinmay M. Trivedi[1,2,4,5,*]

List of Supplementary Materials

Supplemental figures 1 to 4

Supplemental table 1

Supplemental Figure S1

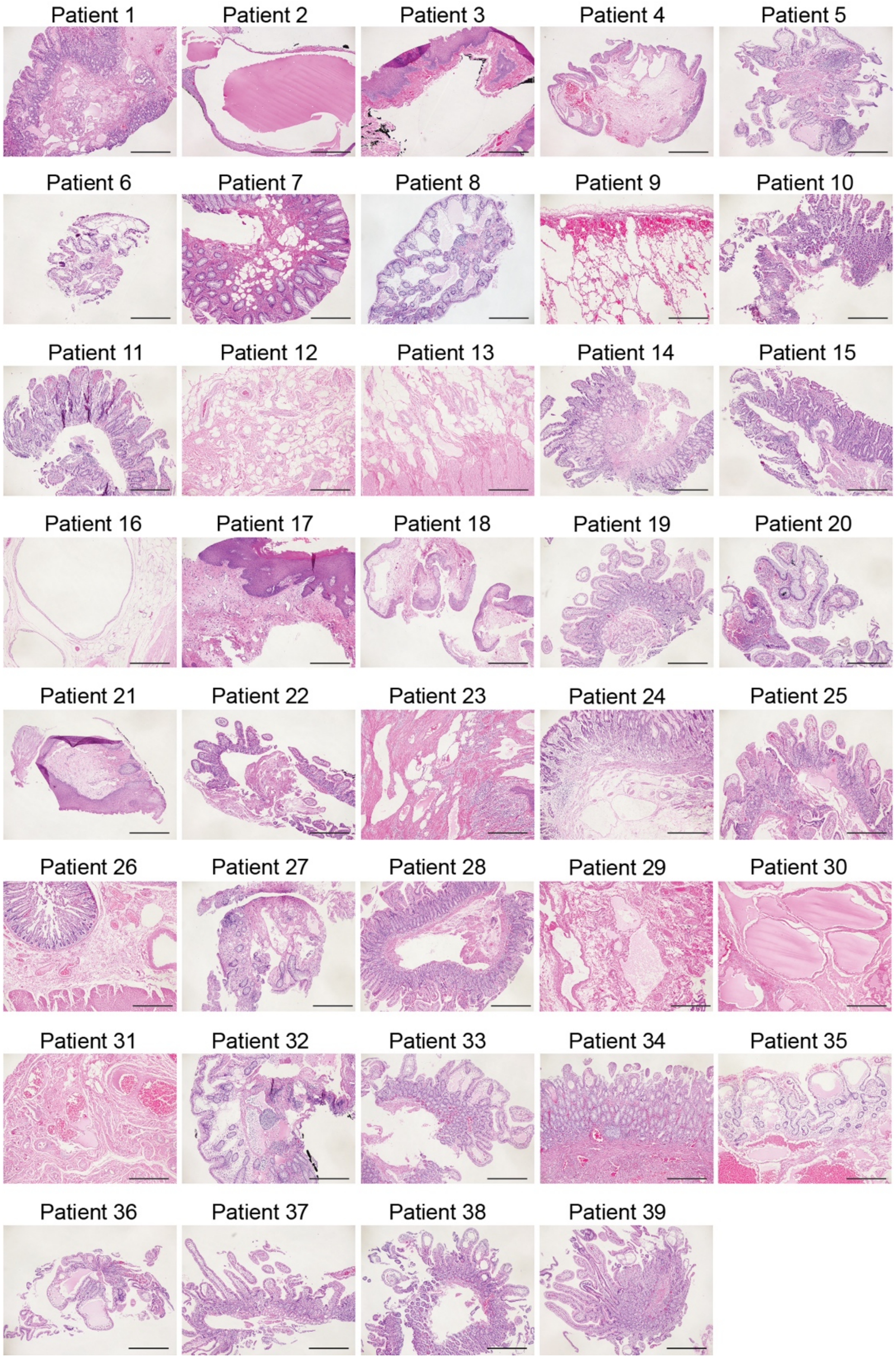

Supplemental Figure S1. Characterization of human lymphangiectasia tissue samples: Hematoxylin & Eosin-stained pathological human tissue section shows lymphangiectasia (n=39). Scale bar 500μm.

Supplemental Figure S2

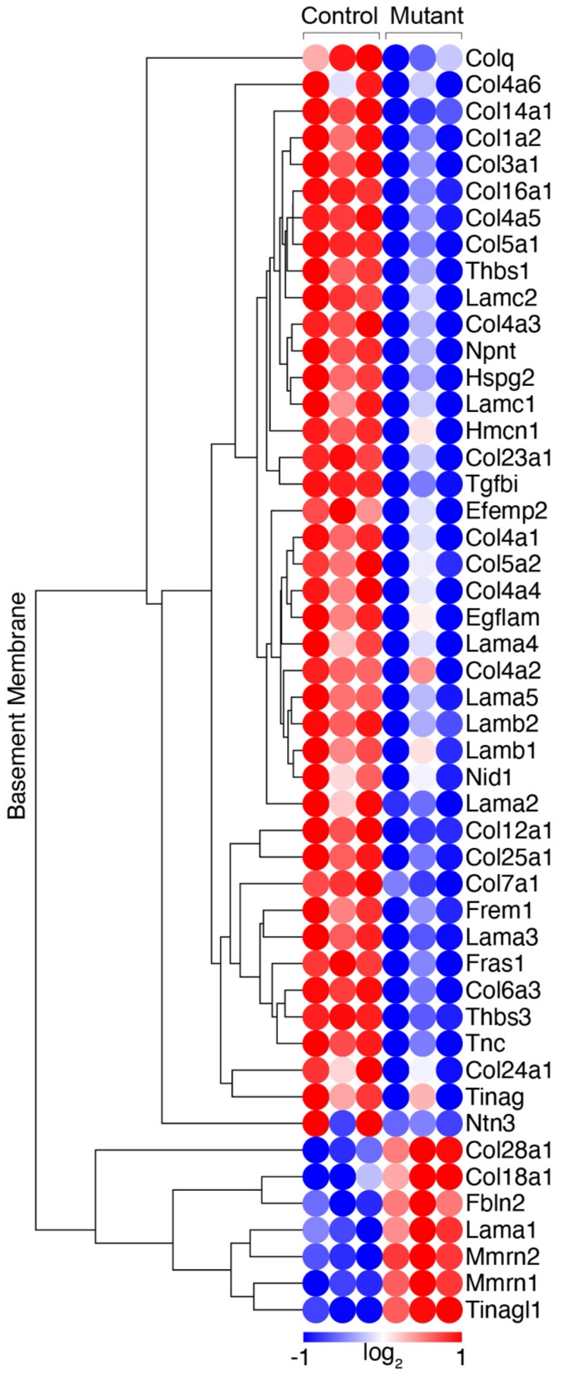

Supplemental Figure S2. Pathological Mapk activation downregulates basement gene transcription. Heatmap of top differentially regulated transcripts within the pathway categories of basement membrane in P12 Kras$^{G12D\ F/+}$; Cdh5^{CreERT2} lungs treated with Tamoxifen compared with control lungs (n = 3).

Supplemental Figure S3

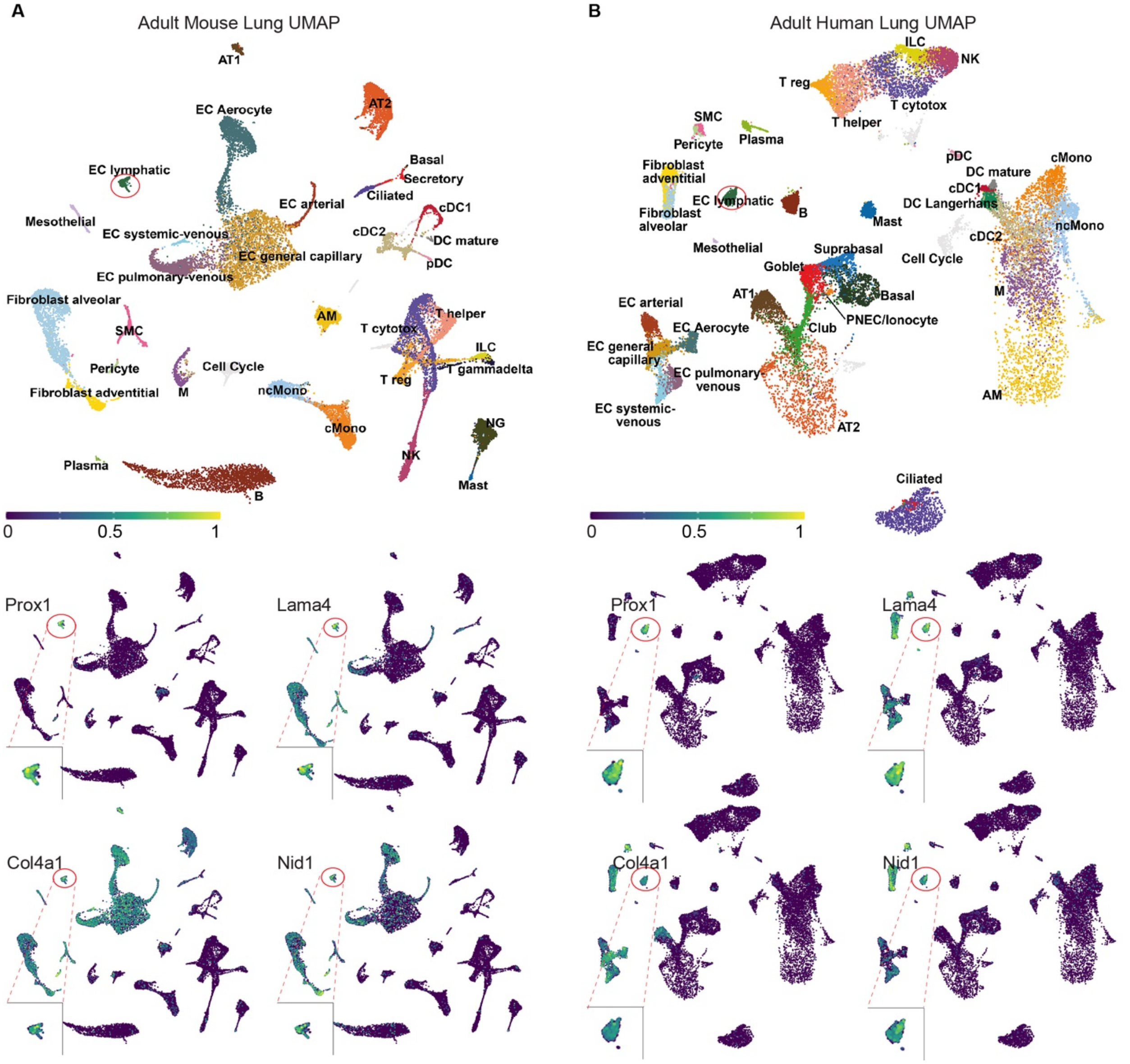

Supplemental Figure S3. Basement membrane gene expression in human and murine lungs at a single cell level: (A-B) Uniform manifold approximation and projection representation of the lung cell dataset (1) of 57,974 cells from 18 control mouse lungs **(A)** and 278,648 cells from 68 control human lungs **(B)**. Each dot represents a single cell. Basement membrane genes show robust expression in Prox1$^+$ lymphatic endothelial cells (red oval). Green to yellow color dot represent higher expression of gene transcript. Purple color dot represent low to no expression of gene transcript. AM, alveolar macrophage; AT1/2, alveolar cell type 1/2; cDC1/2, classical dendritic cell type 1/2; cMono, classical monocyte; DC, dendritic cell; ILC, innate lymphoid cell; M, macrophage; ncMono, nonclassical monocyte; NK, natural killer; pDC, plasmacytoid dendritic cell; PNEC, pulmonary neuroendocrine cell; and SMC, smooth muscle cell.

Supplemental Figure 4

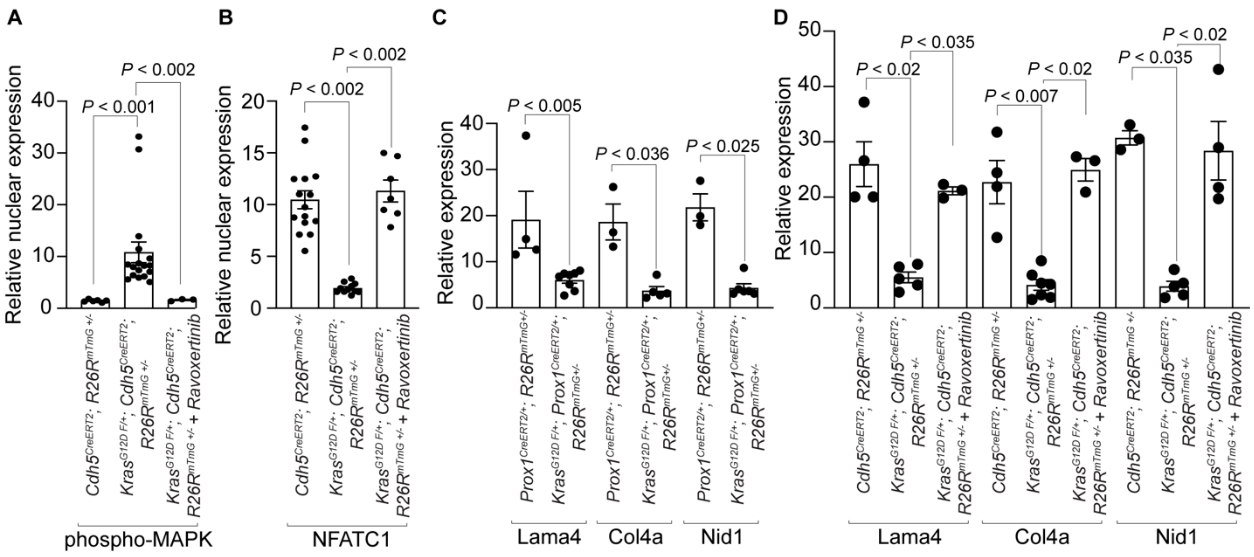

Supplemental Figure S4. (A-B) Quantification of nuclear phosphorylated-MAPK protein (A) or nuclear NFATC1 protein (B) expression relative to Hoechst nuclear staining in intercostal lymphatic vessels of *Kras*$^{G12D\ F/+}$*; Cdh5*^{CreERT2} mutant mice treated with or without Ravoxertinib. **(C-D)** Quantification of relative protein expression in pulmonary and intercostal lymphatic vessels of *Kras*$^{G12D\ F/+}$*; Prox1*^{CreERT2} (C) and *Kras*$^{G12D\ F/+}$*; Cdh5*^{CreERT2} treated with or without Ravoxertinib (D) mutant mice. Data represent the mean ± SEM. *P* values were determined by unpaired non-parametric Mann-Whitney test.

Supplemental Table S1. Antibodies used

Antibody	Company	Catalogue number	Species	Dilution
Lyve1	R&D Systems	AF2125	Goat	1:100
Prox1	Angiobio	11-002	Rabbit	1:100
GFP	Santa Cruz	sc-9996	Mouse	1:100
VE-Cadherin	R&D Systems	AF1002	Goat	1:100
Ki-67	abcam	ab15580	Rabbit	1:100
VegfR3	R&D Systems	AF743	Goat	1:100
Pdpn	DSHB	8.1.1	Hamster	1:100
SMA	abcam	ab5694	Rabbit	1:100
SMA	Sigma	A2547	Mouse	1:133
Collagen IV	Novus Bio	NB120-6586	Rabbit	1:200
Entactin/NID	abcam	ab254325	Rabbit	1:100
Laminin a4	R&D Systems	AF3837	Goat	1:100
Laminin a4	Novus Bio	NBP2-45582	Mouse	1:50
phospho-MAPK	Cell signaling	4370	Rabbit	1:50
Nfatc1	Cell signaling	8032	Rabbit	1:100
Nfatc1	Novus Bio	NB100-56732	Rabbit	1:50
IgG	Cell signaling	66362	Rabbit	1:10
Lyve1	R&D Systems	AF2089	Goat	1:100
Pdpn	Sigma	322M-1	Mouse	1:25
Phospho-Histone H3	Cell signaling	9706	Mouse	1:100
GFP	Santa Cruz	sc-8334	Rabbit	1:100
DyLight 488	Life Technologies	SA5-10086	Donkey	1:500
Alexa Fluor 488	Life Technologies	A-21202	Donkey	1:500
Alexa Fluor 488	Life Technologies	A-21206	Donkey	1:500
Anti-Hamster FITC	Life Technologies	31587	Rabbit	1:500
Alexa Fluor 546	Life Technologies	A-10036	Donkey	1:500
Alexa Fluor 568	Life Technologies	A-11057	Donkey	1:500
Alexa Fluor 568	Life Technologies	A-10042	Donkey	1:500
Alexa Fluor 568	Life Technologies	A-11079	Rabbit	1:500
Alexa Fluor 647	Life Technologies	A-31573	Donkey	1:500
Alexa Fluor 647	Life Technologies	A-21447	Donkey	1:500
Alexa Fluor 647	Life Technologies	A-32787	Donkey	1:500

Reference:

1. Schupp JC, Adams TS, Cosme Jr C, Raredon MSB, Yuan Y, Omote N, et al. Integrated Single Cell Atlas of Endothelial Cells of the Human Lung. *Circulation.* 2021.
